# The G16319A substitution frequency in a hemorrhagic stroke

**DOI:** 10.4103/0972-2327.42934

**Published:** 2008

**Authors:** Barbara Gaweł, Joanna Głogowska-Ligus, Urszula Mazurek

**Affiliations:** Department of Neurology, Medical University of Silesian, Katowice, Poland; 1 Department of Molecular Biology and Genetics, Medical University of Silesian, Katowice, Poland

**Keywords:** Brain stroke, genetic risk factors, mtDNA

## Abstract

**Background:**

The aim of this paper is to trace the nucleotide alterations within the D-loop region of the mitochondrial DNA, affecting both the mtDNA ability to replicate and the transcription activity of the coding genes located in the H and L threads, in Caucasian patients with an ischemic and hemorrhagic brain strokes.

**Materials and Methods:**

The DNA from the peripheral blood of 85 patients with recent sustained ischemic and primary hemorrhagic brain stroke was analysed. The control group consisted of 24 volunteers. The genetic studies were conducted by the PCR method, with the application of the primers for the tRNA-treonine.

**Results:**

In the blood samples examined, 3-striatal mtDNA patterns were detected. Pattern-1 is characterised by the C16126T substitution, pattern-2 by the G16319A substitution, and pattern-3 by the C16242T substitution. The frequency of occurrence for the particular mtDNA-1, -2, and -3 patterns, established for the group with an ischemic stroke (77.3, 15.2, and 7.6%), the group with a hemorrhagic stroke (0, 73.7, and 26.3%), and the control group (75, 0, and 25%), differs significantly.

**Discussion:**

The exchange of the nucleotides within the D-loop region may affect both the mtDNA replication ability and the transcription activity of the coding genes located in the H and L threads. A hypothesis might be made. The G16319A mutation may result in the formation of lesions within the vascular wall. These lesions have a tendency to form microaneurysms or other defects, which, in turn, will decrease the strength of the vascular wall, making it more susceptible to ruptures.

**Conclusion:**

The G16319A substitution may be considered a factor that increases the risk of a hemorrhagic brain stroke.

## Introduction

The results of many epidemiological studies, as well as studies on twins, indicate a significant effect of genetic factors on the incidence of brain strokes. In the evaluation of strokes as familial disorders, nongenetic factors such as lifestyle, including similar nutrition habits, alcohol consumption, physical activity, and smoking, should be carefully considered. The nongenetic background of strokes may be a consequence of mutations of single genes[1] or it may be the result of the activity of many mutated genes, along with the effect of environmental factors.[2]

In recent years, a new category of genetic disorders caused by defects of the mitochondrial genome has been described. The most common disorders of the nervous and the muscular tissues caused by a damage to mtDNA are: MERFF,[3] MELAS[4] and Kearns-Saye's[5] and Leigh's[6] syndromes. Mitochondrial encephalomyopathy, lactic acidosis, and stroke (MELAS) are characterized by the A3243G mutation of the tRNA-leu gene. However, only 50% of patients with the A3243G mutation suffer a stroke. Other phenotypes include myopathy, chronic progressive extermal ophthalmoplegia, diabetes mellitus, and deafness.[7]

The aim of this paper is to trace the alterations in the D-loop region of the mitochondrial DNA in patients with ischemic and hemorrhagic brain strokes, and to establish the correlation between the type of brain stroke and the alteration in mtDNA. (The D-loop region is a long noncoding nucleotide sequence of the mtDNA, which is the most variable portion of replication and transcription control of the mtDNA.) The group of persons with hemorrhagic brain stroke comprised only those with primary haemorrhage.

## Materials and Methods

The material for the study as well as the data on the disorder was collected from the patients treated in the Department of Neurology of the Medical University of Silesia in Katowice. Peripheral blood DNA of the patients enrolled in the group was analysed. Brain stroke was diagnosed on the basis of the focal neurological symptoms of duration over 24 hours, confirmed by radiological examinations such as CT scan or magnetic resonance.

Eighty five patients, including 33 females and 52 males aged 37-70 (average age 59.9), were examined. The control group consisted of 24 volunteers - 9 females and 15 males, aged 35-69 (average age 56.1), randomly selected from the home area of the patients with a brain stroke. All patients under examination belonged to the Caucasian race.

The condition for enrolment in the control group was negative past history of neurological disorders, particularly lack of previous sustained brain strokes and lack of any syndromes for transient brain ischemia. In the clinical researches, risk factors such as diabetes mellitus, arterial hypertension, atrial fibrillation, and coronary arterial disease were considered. Before being admitted to the clinical group, full biochemical, coagulated and hematological tests were done on all the patients. After receiving consent from the Bioethical Committee of Silesian Medical University, the study began on June 7, 2001.

“Blood DNA Prep Plus” kit, manufactured by A and A Biotechnology, was used for the isolation of DNA from the study material. The DNA was used directly for testing, without the need to cause precipitation. The evaluation of mtDNA variability was conducted by means of the PCR technique, using suitable primers for domain of mtDNA D-loop:

H2 (H16401)5′-TGATTTCACGGAGGATGGTG-3′;
L1 (L15926tRNA-Thr): TCAAAGCTTACACCAGTCTTGTAAAAC;
L2 (L15997tRNA-Pro): CACCATTAGCACCCAAAGCT”

The amplification was performed the in GeneAmp PCR System 9600, manufactured by Perkin-Elmer (Massachusetts, USA), in the following temperature conditions: initial denaturation − 94°C, second denaturation at; 92°C - 45 seconds; annealing at 48°C - 45 seconds; extension at 72°C - 1 minute 30 seconds and final elongation: 72°C - 5 minutes. The PCR reaction was conducted in a reaction mixture containing 20× reaction buffer for Tfl, 25 mM MgCl2, 10× amplification intensifier, dNTP (of 2.5 mM concentration each), starters complementary to tRNA-Thr, apyrogenic water, and the enzyme Tfl polymerise 1U/l. The obtained amplimers were subsequently separated, using the Multitemperature Single Strand Conformation Polimorphism (MSSCP) technique, in 8% polyacrylamide gel with glycerol. The gel was coloured with the silver method. The outcomes received by the MSSCP technique were confirmed through PCR analysis, by using ABI PRISM 700 Sequence Detection System, with the help of the Quanti Tect^TM^ SYBR Green PCR Kit. The final stage of analysis of the polymorphism mtDNA was performed by using Seq Scape programme Version 1.1 (Applied Bio systems USA).

**Figure 1:**
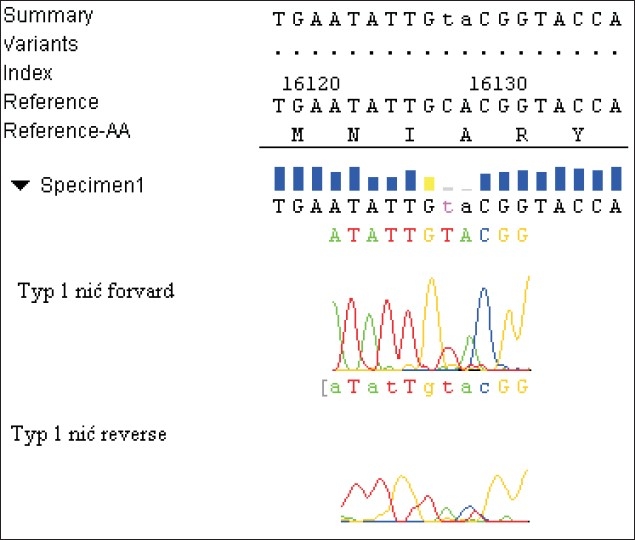
Substitution characteristic for pattern-1 C16126T. Exchange of cytosine for thymine in the position of 16126 base pairs

**Figure 2:**
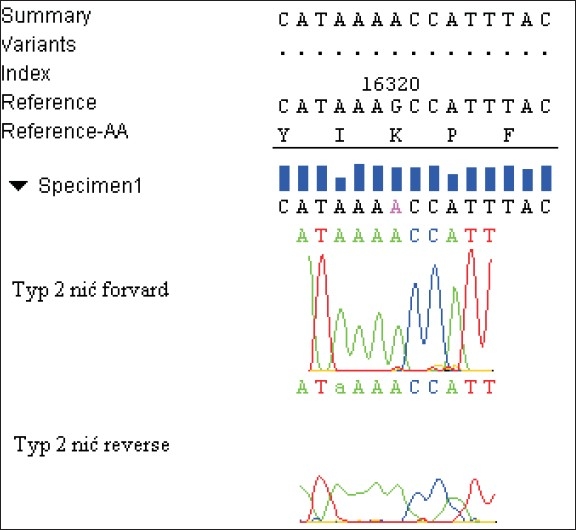
Substitution characteristic for pattern-2 G16319A. Exchange of guanine for adenine in the position of 16319 base pairs

**Figure 3:**
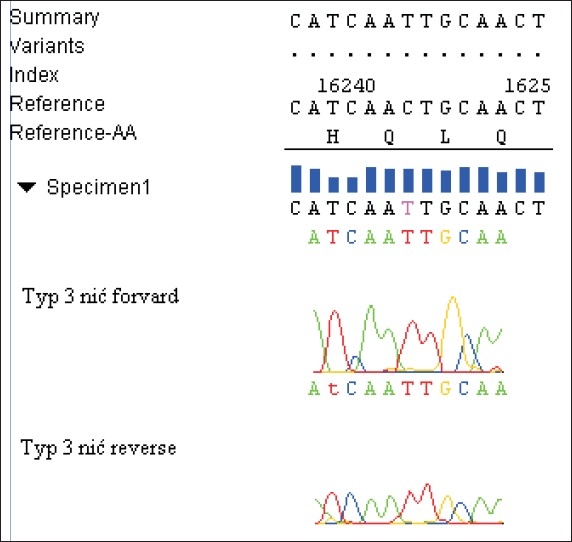
Substitution characteristic for pattern-3 C16242T. Exchange of cytosine for thymine in the position of 16242 base pairs

Three dominant different patterns of nucleotides sequence, referred to as the polymorphism types, were obtained, each of them with a characteristic substitution for a given type, but different from the Homo Sapiens Haplotype As 9Y Mitochondrian, complete genome [Gi: 29691050] bank sequence. In type-1, an exchange in the forward thread of cytosine in the position 16126 base pairs for thymine (C16126T) was demonstrated. In type-2, an exchange of guanine in the position of 16319 base pairs for adenine (G16319A) occurred. In type-3, a substitution of cytosine by thymine in 16242 base pairs (C16242T) was found [Figures 1–3].

Other substitutions, different from the [Gi: 29691050] bank sequence were also detected. The three common ones, such as (C16189T) and (C16231T) and (T16266C), were found in all the persons under examination. The additional common substitution (C16096T) found in all patients presented substitutions such as: (G16319) and (C16242T).

Statistical evaluation was performed with regard to the number of the researched persons in the ischemic and hemorrhagic brain strokes group and in the control group. All the patients had their data recorded in the EXCEL calculation sheet computer program. The frequency rate for the particular polymorphism types of the mitochondrial DNA, among the researched groups, was compared. The statistical analysis of the results was performed at the significance level *P*=<0.05. The hypothesis advocating a higher frequency (statistically higher fraction) for risk factors or for particular genetic lesions was verified by comparing the two populations in whom the analysed factor was present: the group with a stroke and the control group, or the group with an ischemic stroke and the group with a hemorrhagic stroke. An analysis was conducted in order to verify the hypothesis on the equality of the fraction indices for the two populations with a normal standardized distribution N (0.1). Additionally, based on the Chi-square independence test, the hypotheses on the dependence of brain stroke occurrence on the observed polymorphism types were verified.

## Results

On examining the 109 blood samples, including those from 66 patients with an ischemic stroke and 19 with a hemorrhagic brain stroke, three different patterns of nucleotides sequence mtDNA were detected.

**Figure 4:**
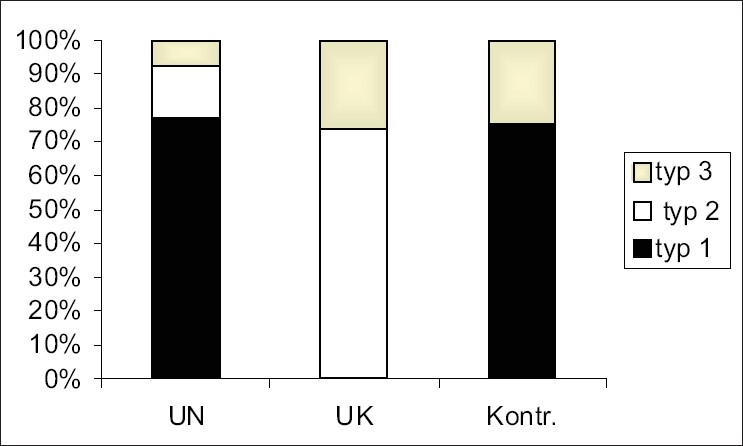
Prevalence of mtDNA polymorphism in the ischemic stroke, hemorrhagic stroke and control group. The gels were stained using the silver method – str. 4

The respective frequency values for the particular polymorphic type were as follows:

- In the ischemic stroke group, pattern-1: 51 patients (77.3%); pattern-2: 10 patients (15.1%); pattern-3: 5 patients (7.6%)
- In the hemorrhagic stroke group, pattern-1: absent; pattern-2: 14 patients (73.7%); pattern-3: 5 patients (26.3%)
- In the control group, pattern-1: 18 persons (75%); pattern-2: absent; pattern-3: 6 (25%) [Table 1, Figure 4].

**Table 1:** The number and percentage of patients with focus on the type of mtDNA polymorphism

mtDNA	The number of patients and the percentage value (%)			
	
	Control	Patients	Ischemic stroke	Hemorrhagic stroke
Type-1	18 (75)	51 (60)	51 (77,3)	0
Type-2	0	24 (28,2)	10 (15,1)	14 (73,7)
Type-3	6 (25)	10 (11,8)	5 (7,6)	5 (26,3)
Total	24 (100)	85 (100)	66 (100)	19 (100)

Figures in parentheses are in percentage

The frequency values for type-1 and type-3 alterations in the control group and in the brain stroke group do not show statistically significant differences. Type-2 alteration occurs significantly more frequently (p<0.0001) in the brain stroke group, as compared to the control. The frequency values for the particular mtDNA-1, -2, -3 patterns in the group of patients with an ischemic brain stroke (77.3, 15.2, and 7.6%, respectively) and in the patients with a hemorrhagic stroke (0, 73.7, and 26.3%, respectively) differ significantly.

Type-1 alteration in mtDNA is present solely in an ischemic stroke; it is not observed in a hemorrhagic stroke. Type-2 occurs significantly more frequently (p<0.0001) in a hemorrhagic stroke; also, the percentage of type-3 is three times higher in a hemorrhagic stroke (p<0.05). There is no statistically significant difference with regard to type-1 mtDNA alteration occurrence rate in the ischemic stroke group (77.3, 15.2 and 7.6%, respectively) in comparison with the control group (75, 0 and 25% respectively) (p<0.05). However, there are statistically significant differences in the frequency rates for type-2 and type-3 (p<0.05). The absence of type-2 in the control group has been noted.

A statistically significant effect of the presence of type-1 (p<7.39 × 10^7^) and type-2 (p<3.04 × 10^7^) mtDNA lesions, on the occurrence of an ischemic stroke, has been observed. No significant effect of the presence of type-3 mtDNA alteration upon the occurrence of a hemorrhagic stroke has been demonstrated. Thus, there is a statistically significant effect of the presence of type-1 mtDNA alteration on the type of brain stroke (p<1.4 × 10^9^).

## Discussion

In 1988, the first mutation of the mitochondrial DNA was identified. In 1990, only three such mutations were known, whereas in 2001, it was as high as 70.[8] Apart from the numerous pathogenic mtDNA mutations, a great number of polymorphism of the mitochondrial DNA, which are not pathogenous at all, have been identified. They are common among the members of a given population or ethnic group.[9] This polymorphism may indicate the origin of a particular community group and its protoplasts.

The D-loop region of mtDNA is the most interesting for research, because it contains the control region for replicating the initiation of H-stand and L-stand. Destabilised D-loop structure inhibiting replication leads to a decrease in the number of mtDNA and disturbs the right sequence gene and the H-strand and L-strand transcription.[10] Mutations in the D-loop region have been responsible for the transcription activity of the genes coded in the mtDNA. Additionally, the mentioned D-loop affects the energetic state of the cell during the proteins' production of the complex respiratory chain. An insufficient number of highly energetic ATP compounds disturbs the function of the metabolic pathways of the cell, which leads to death of this cell.[11]

The consequences of tissue ischemia, resulting from a limited oxygen and glucose supply, are the same as in the case of damage to the mitochondrial respiratory chain, i.e. insufficient number of the highly energetic ATP compounds.[12] In 2004, Liou CW has suggested that the mtDNA T16189C variant is a predisposing genetic factor for the development of insulin resistance and may be related to various phenotypic expressions in adult life, such as development of diabetes mellitus (DM) and vascular pathologies involved in stroke and cardiovascular diseases.[13] In 2005, Lin TK also reported that transition of T to C at nucleotide position 16189 in mtDNA has attracted biomedical researchers for its probable correlation with the development of diabetes mellitus (DM) in adult life. In diabetes and persistent hyperglycemia, it may cause a high production of free radicals.

Reactive oxygen species are thought to take part in a variety of physiologic and pathophysiologic processes in which increased oxidative stress may play an important role in disease mechanisms.[14] The T16189C variant of mtDNA is not only present in Caucasians, but also in the Chinese subject.[15] These information might suggest that the polymorphic type in D-loop region is perhaps related to a concrete mutation of a gene which is situated in the H or L-strand.

Our research has demonstrated that type-2 alteration in the mtDNA, found in the G16319A substitution in the D-loop region, is significantly more frequent in a hemorrhagic stroke, in which sudden extravasations of blood due to a rupture of the blood vessel occurs.

The exchange of the nucleotides within the D-loop region may affect both the mtDNA replication ability and the transcription activity of the coding genes located in the H and L threads.[16,17] Having associated these two facts, a hypothesis may be made that the G16319A mutation may result in the formation of lesions within the vascular wall, which cause a tendency to form microaneurysms or other defects, which, in turn, decrease the strength of the vascular wall, making it more susceptible to ruptures.

Further studies conducted on a larger population are advisable in order to either confirm or reject this hypothesis. We think that the search for genes' mutations of the H- and L-strand or their expression is applicable to the polymorphic type of D-loop region, which could prove to be a background for explaining the hypothesis. It would also be helpful in discovering the mechanism by which the abnormal mtDNA genetic code contributes to the formation of a somatic defect.

## Conclusion

The G16319A (type-2) substitution may be considered a genetic factor that increases the risk of a hemorrhagic brain stroke.

## References

[1] Dong Y, Hassan A, Zhang Z, Huber D, Dalageorgou C, Markus HS (2003). Yield of screening for CADASIL mutations in lacunars stroke and leukoaraiosis. Stroke.

[2] Nguyen LT, Ramanathan M, Weinstock-Guttman B, Baier M, Brownscheidle C, Jacobs LD (2003). Sex differences in vitro pro-inflammatory cytokine production from peripherical blood of multiple sclerosis patients. J Neurol Sci.

[3] Hammans SR, Sweeney MG, Brockington M, Lennox GG, Lawton NF, Kennedy CR (1993). The mitochondrial DNA transfer RNA^Lys^ A⇒G^(8344)^ mutation and the syndrome of myoclonic epilepsy with ragged red fibres (MERRF): Relationship of clinical phenotype to proportion of mutation mitochondrial DNA. Brain.

[4] Kolb SJ, Costello F, Lee AG, White M, Wong S, Schwartz ED (2003). Distinguishing ischaemic stroke from the stroke-like lesions of MELAS using apparent diffusion coefficient mapping. J Neurol Sci.

[5] Seneca S, Verhelst H, De Meirleir L, Meire F, Ceuterick-De Groote C, Lissens W (2001). A new mitochondrial point mutation in the transfer RNA^Leu^ gene in a patient with a clinical phenotype resembling Kearns-Sayre syndrome. Arch Neurol.

[6] Moslemi AR, Tulinius M, Darin N, Aman P, Holme E, Oldfors A (2003). SURF 1 gene mutations in three cases with Leigh syndrome and cytochrome c oxidase deficiency. Neurology.

[7] Pulkes T, Sweeney MG, Hanna MG (2000). Increase risk of strokes in patients with the A12308G polymorphism in mitochondria. Lancet.

[8] Florentz C, Sissler M (2001). Disease-related versus polymorphic mutation in human mitochondrial tRNAs. Eur Molec Biolog Org [EMBO reports].

[9] Finnila S, Lehtonen SM, Majamaa K (2001). Phylogenetic network for European mtDNA. Am J Hum Genet.

[10] Bielawski JP, Gold JR (2002). Mutation patterns of mitochondrial H- and L-strand DNA in closely related cyprinid fishes. Genetics.

[11] Sastre J, Pallarido FV, de la Asuncion GJ, Vina J (2000). Mitochondria, oxidative stress and aging. Free Radiat Res.

[12] Lee JM (2001). Neuronal cell death in nervous system development, disease, and injury. (Review). Int J Nucl Med.

[13] Liou CW, Lin TK, Huang FM, Chen TL, Lee CF, Chuang YC (2005). Association of the mitochondrial DNA 16189 T to C variant with Lacunar cerebral infarction: Evidence from a hospital-based case-control study. Ann NY Acad Sci.

[14] Lin TK, Chen SD, Wang PW, Wei YH, Lee CF, Chen TL (2005). Increasd oxidative damage with altered antioxidative status in type 2 diabetic patients harboring the 16189 T to C variant of mitochondrial DNA. Ann N Y Acad Sci.

[15] Weng SW, Liou CW, Lin TK, Wei YH, Lee CF, Eng HL (2005). Association of mitochondrial deoxyribonucleic acid 16189 variant (T->C transition) with metabolic syndrome in Chinese adults. J Clin Endocrinol Metab.

[16] Mishmar D, Ruiz-Pesini E, Golik P, Macaulay V, Clark AG, Hosseini S (2003). Natural selection shaped regional mtDNA variation in humans. Proc Natl Acad Sci USA.

[17] Pluzhnikov A, Di Rienzo A, Hudson RR (2002). Inferences about human demography based on multilocus analyses of noncoding sequences. Genetics.

